# Supplementary material for: Equity Impact Assessment of Interventions to Promote Physical Activity among Older Adults: A Logic Model Framework
Source: Int J Environ Res Public Health. 2019 Feb 1;16(3):420. doi: 10.3390/ijerph16030420 (PMC6388141; doi:10.3390/ijerph16030420)
Supplement: Supplementary file 1 [file ijerph-16-00420-s001.pdf]

Supplementary File

Table S1. Interview guidelines for researchers and practitioners on social inequalities-sensitive physical activity promotion in older adults.

Interview questions for researchers	Interview questions for practitioners
- Which groups of older adults warrant special attention in health promotion and prevention?<br>- Which groups of older adults are reached by existing measures of physical activity promotion/health promotion and prevention, and which groups are hardly reached?<br>- Are there strategies that have proven to be successful in reaching different groups of older adults?	- Which groups of older adults do you want to reach?<br>- How do you define 'socioeconomically disadvantaged' older adults?<br>- How did you choose these groups?<br>- Which actors were involved in the selection of these groups?<br>- Which groups of older adults have actually been reached?<br>- Which strategies have proven to be successful in reaching these groups?<br>- Are there differences between different groups?<br>- Which strategies have proven to be less effective in reaching these groups?<br>- Are there groups of older adults that have hardly been reached so far?<br>- If yes: What do you think why these groups have hardly been reached so far?
- How to consider differences related to gender, socioeconomic status, or a migration background in the development of interventions?	- Do you use intervention methods to consider differences related to gender, socioeconomic status, or a migration background?
- Where do you see success in reaching socioeconomically disadvantaged groups, and where do you see gaps? How would you rate the current evidence?<br>- What are indicators for measuring this success?<br>- Do you have practical experience in evaluating equity impacts of interventions ('equity impact assessment')?<br>- If yes: What methods for evaluation did you use?<br>- If no: Imagine you have developed an intervention and now want to investigate the impact of this intervention on social inequalities: What methods for evaluation would you use?	- How are your offers evaluated?<br>- How do you assess the effectiveness of your offers?<br>- Are there differences in the effectiveness between different groups, such as between men and women, individuals with low and high socioeconomic status, or individuals with and without a migration background?<br>- If yes: What do you think why these differences occurred?
